# Insect antimicrobial peptides show potentiating functional interactions against Gram-negative bacteria

**DOI:** 10.1098/rspb.2015.0293

**Published:** 2015-05-07

**Authors:** Mohammad Rahnamaeian, Małgorzata Cytryńska, Agnieszka Zdybicka-Barabas, Kristin Dobslaff, Jochen Wiesner, Richard M. Twyman, Thole Zuchner, Ben M. Sadd, Roland R. Regoes, Paul Schmid-Hempel, Andreas Vilcinskas

**Affiliations:** 1 Department of Bioresources, Fraunhofer Institute for Molecular Biology and Applied Ecology, Winchester Strasse 2, Giessen 35394, Germany; 2 Department of Immunobiology, Institute of Biology and Biochemistry, Maria Curie-Sklodowska University, Akademicka Street 19, Lublin 20–033, Poland; 3 Institute of Bioanalytical Chemistry, Faculty of Chemistry and Mineralogy and Center of Biotechnology and Biomedicine, University of Leipzig, Deutscher Platz 5, Leipzig 04103, Germany; 4 TRM Ltd, PO Box 93, York YO43 3WE, UK; 5 School of Biological Sciences, Illinois State University, Campus Box 4120, Normal, IL 61790, USA; 6 ETH Zürich, Institute of Integrative Biology, ETH-Zentrum CHN, Universitätsstrasse 16, Zürich 8092, Switzerland; 7 Institute of Phytopathology and Applied Zoology, Justus-Liebig-University of Giessen, Heinrich-Buff-Ring 26–32, Giessen 35392, Germany

**Keywords:** antimicrobial peptides, innate immunity, insects, abaecin, hymenoptaecin, bumble bees

## Abstract

Antimicrobial peptides (AMPs) and proteins are important components of innate immunity against pathogens in insects. The production of AMPs is costly owing to resource-based trade-offs, and strategies maximizing the efficacy of AMPs at low concentrations are therefore likely to be advantageous. Here, we show the potentiating functional interaction of co-occurring insect AMPs (the bumblebee linear peptides hymenoptaecin and abaecin) resulting in more potent antimicrobial effects at low concentrations. Abaecin displayed no detectable activity against *Escherichia coli* when tested alone at concentrations of up to 200 μM, whereas hymenoptaecin affected bacterial cell growth and viability but only at concentrations greater than 2 μM. In combination, as little as 1.25 μM abaecin enhanced the bactericidal effects of hymenoptaecin. To understand these potentiating functional interactions, we investigated their mechanisms of action using atomic force microscopy and fluorescence resonance energy transfer-based quenching assays. Abaecin was found to reduce the minimal inhibitory concentration of hymenoptaecin and to interact with the bacterial chaperone DnaK (an evolutionarily conserved central organizer of the bacterial chaperone network) when the membrane was compromised by hymenoptaecin. These naturally occurring potentiating interactions suggest that combinations of AMPs could be used therapeutically against Gram-negative bacterial pathogens that have acquired resistance to common antibiotics.

## 1. Introduction

The ability of multicellular organisms to defend themselves against microbes is mediated by their immune systems. Whereas higher vertebrates possess both innate immunity and an adaptive arm of the immune system based on expanding B cell and T cell populations with specificity towards particular antigens, insects and most other animals rely on the evolutionarily more ancient innate immune system. When the innate immune system is activated, it produces a broad spectrum of effector molecules including antimicrobial peptides (AMPs) [1]. The latter play multifaceted roles in insects including the killing of bacteria that survived constitutive defences such as phagocytosis and multicellular encapsulation [2]. Comparative genomics and transcriptomics have demonstrated the remarkable evolutionary plasticity of insect immunity in terms of the rapid gain, loss and functional shifting of AMPs [3,4].

AMPs have diverse modes of action, e.g. by changing the transmembrane electrochemical gradients necessary for microbial homeostasis, inhibiting protein synthesis, inducing membrane permeabilization and rupture, or promoting the synthesis of reactive oxygen species that cause cell death [5–6]. The number of AMPs in insects varies considerably among different species, ranging from more than 50 in the invasive ladybird *Harmonia axyridis* [7] to a lack of any known antibacterial AMPs in the pea aphid *Acyrthosiphon pisum* [8]. The honeybee *Apis mellifera* produces only six AMPs, which is unexpected considering the genetic similarity among bees in a hive and their close contacts, meaning that even the exchange of food risks the rapid spread of pathogens vectored by workers from outside [9,10].

We considered the possibility that functionally distinct insect AMPs may act together when expressed simultaneously during an innate immune response. The possibility that some AMPs may primarily act to permeabilize or destroy the bacterial membrane to facilitate the activity of other components of the immune system has been raised before [11], but work has often focused on the synergistic effects of non-natural combinations [12–14]. By contrast, the synergistic/potentiating actions among naturally co-occurring and co-expressed AMPs in insects have received little attention [15–17] compared with vertebrates [18–26]. Insect AMPs are indeed co-expressed [27–29], and naturally co-occurring AMPs display potentiating effects on bacterial pathogens [15,16].

Beneficial AMP interactions may be achieved by synergism (greater than additive antimicrobial effects), potentiation (one AMP enabling or enhancing the activity of others) and functional diversification, i.e. combinatorial activity increasing the spectrum of responses and thus the specificity of the innate immune response, perhaps even to rival the specificity of adaptive immune systems [29–31]. This may enable the direct targeting of specific pathogens, increase the efficacy and robustness of antimicrobial responses, and ultimately reduce the resources committed to the innate immune system by increasing the antimicrobial activity of AMPs at low concentrations [2,7,32–34].

Insect AMPs can be assigned to different classes according to their molecular structure and/or the presence of particular amino acid residues [1,35]. For example, proline-rich AMPs are characterized by abundant proline residues and have two domains, one conserved domain responsible for general antimicrobial activity and one variable domain conferring microbial specificity. The short-chain AMPs in this class (fewer than 20 residues) primarily target Gram-negative bacteria, whereas their long-chain counterparts (more than 20 residues) mainly affect Gram-positive bacteria and fungi [36–39]. Thus far, proline-rich AMPs have been characterized in the Hymenoptera, Diptera, Hemiptera and Lepidoptera [40]. They can interact with the 70S ribosome and thereby inhibit protein biosynthesis [41], and with DnaK, an evolutionarily conserved central organizer of the bacterial chaperone network, abolishing its ability to mediate chaperone-assisted protein folding and ribosomal biogenesis [42–45].

Here, we describe a functional interaction between two AMPs from the bumblebees *Bombus pascuorum* Scopoli and *B. terrestris* L [46,47]. The functional significance of these AMPs in the defence against common protozoan parasites has recently been demonstrated using RNAi [48]. We investigated the effects of the glycine-rich peptide hymenoptaecin (identical in both species) and the proline-rich peptide abaecin, differing by one amino acid at position 17 (electronic supplementary material, table S1). Gene expression studies have shown that these peptides are expressed simultaneously and released into the haemolymph during innate immune responses [28,49]. We used a novel computational method to measure the antibacterial activity of the AMPs alone and in combination against the bacterium *Escherichia coli* based on *in vitro* models of bacterial growth and viability, investigated their structural impact on the bacterial cell envelope and their mechanisms of action, and identified a novel sequence that is likely to mediate the activity of abaecin.

## 2. Material and methods

### (a) Microorganisms

We used *E. coli* strains D31 and 498 (Leibniz Institute DSMZ German Collection of Microorganisms and Cell Cultures) and JM83, carrying plasmid pCH110 (Pharmacia-Amersham, Piscatway, NJ, USA).

### (b) Peptide synthesis and modification

A detailed description of the peptide synthesis and modification procedure is provided in the electronic supplementary material.

### (c) Labelling DnaK with BHQ10

DnaK, produced by Michael Zahn [50], was dialysed against modifying buffer (20 mmol l^−1^ Na_2_HPO_4_, 20 mmol l^−1^ KH_2_PO_4_, 5 mmol l^−1^ MgCl_2_, 150 mmol l^−1^ KCl, pH 7.4) as previously described [51] and 2 mg ml^−1^ were labelled with a 10-fold molar excess of BHQ10-NHS-ester [52], followed by supplementary dialysis to remove excess BHQ10. The labelling efficacy was determined by measuring absorption at 515 nm, and the labelling ratio was 1 : 9 DnaK : BHQ10.

### (d) Fluorescence resonance energy transfer assay

At the 1 : 9 labelling ratio stated above, the binding of peptides to *E. coli* DnaK resulted in quenching effects detected as a reduction in the intensity of fluorescein emission. As previously described [51], the fluorescein-modified peptides (50 μl, 1.3 nmol l^−1^) and a serial dilution of DnaK–BHQ10 in modification buffer (50 μl, 0.8–13 000 nmol l^−1^, 1 : 4 dilution series) were mixed in a black 384-well plate and incubated for 2 h. To calculate the quenching effect, a control was measured in five replicates consisting of 50 μl of peptide solution and 50 μl of modification buffer. The fluorescence intensity was recorded on a Paradigm fluorescence reader using a fluorescence intensity (fluo-rhod) detection cartridge (excitation wavelength = 485 ± 10 nm; emission wavelength = 535 ± 12.5 nm; integration time = 140 ms). The quenching effect was defined as the percentage of the fluorescence intensity of the control quenched after the addition of DnaK–BHQ10. *K*_d_ values were determined as described in the electronic supplementary material.

### (e) *Escherichia coli* permeabilization assay

The membrane permeabilizing activities of AMPs were determined using *E. coli* strain JM83 on the basis of *β*-galactosidase activity leaking from the cytoplasm [53], as described in detail in the electronic supplementary material. Living bacteria incubated with medium only were used as a negative control and bacteria killed by treatment with 5 µM synthetic cecropin B (Sigma-Aldrich) were used as a positive control (100% permeabilization). Before setting the perforation level of the positive control to 100%, the perforation value obtained for the negative control was subtracted from all other measurements. All assays were carried out three times, each time in triplicate. The results were presented as means ± s.d. (*n* = 3) based on statistical analysis using Student's *t*-test.

### (f) Atomic force microscopy imaging of bacterial cells

Bacteria were prepared for imaging as previously described [53,54] (see electronic supplementary material). The data were analysed with Nanoscope Analysis software v. 1.40 (Veeco, USA). Three fields on each mica disc were imaged. Three-dimensional images and section profiles were prepared using WSxM v. 5.0 software [55]. The roughness values were measured over the entire bacterial cell surface on 3 × 3 µm^2^ areas. The average surface root mean square (RMS) roughness was calculated from 25 fields (300 × 300 nm^2^). The data were analysed using Statistica v. 6 (StatSoft, Inc., Tulsa, OK, USA). Statistical significance was determined by ANOVA (Tukey's honestly significant difference test).

### (g) Amino acid sequence analysis

The peptide amino acid sequences were aligned with pyrrhocoricin, oncosin Onc72, apidaecin Api88 and drosocin using ClustalW [56] followed by manual trimming for the improved alignment of proline residues.

### (h) Growth inhibition and cell viability assays

Initial coarse-level growth inhibition assays were carried out with concentrations of peptides up to 200 μM. Further assays of growth rates (bacteriostatic activity) and cell viability (bactericidal activity) were carried out on a finer scale. These assays used a full matrix with final abaecin concentrations of 0, 1.25, 2.5, 5, 10 and 20 μM, and final hymenoptaecin concentrations of 0, 0.625, 1.25, 2.5, 5 and 10 μM. Growth rates were derived from spline-fitted growth curves to optical density (OD) data, while cell viability was assessed by plating cultures and counting colony-forming units after 18 h. Dose–response curves were fitted to both the bacteriostatic and bactericidal data with Markov Chain Monte Carlo parameter optimization (further details in electronic supplementary material).

## 3. Results

### (a) Abaecin and hymenoptaecin show functional interactions

**Figure 1.**
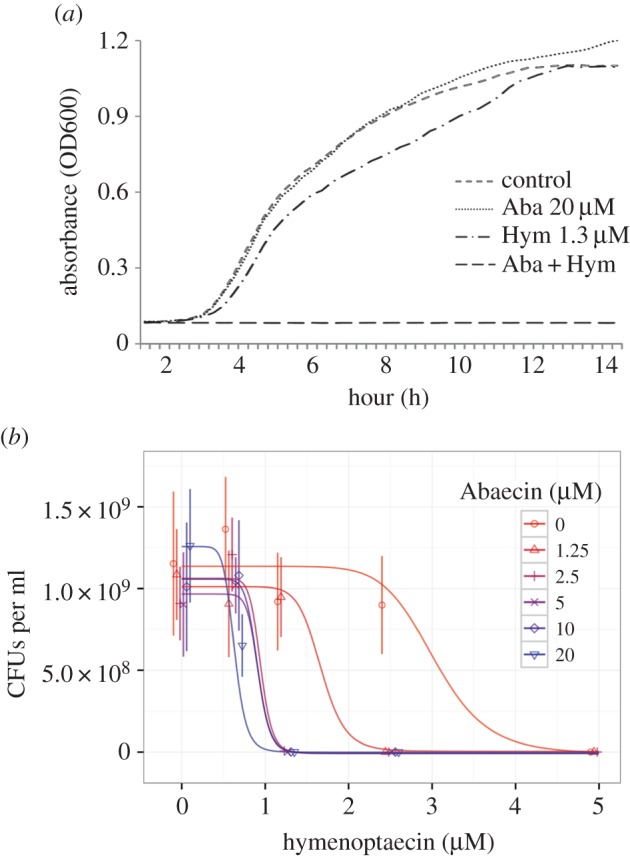
*E. coli* growth inhibition assays. (*a*) *E. coli* strain D31 in mid-logarithmic phase was incubated with medium only (control) or with the concentrations of abaecin (Aba) and hymenoptaecin (Hym) as shown, alone or in combination. The growth rate was determined by measuring the OD of the culture at 600 nm. (*b*) Fitted dose-response curves for *E. coli* viability measured by determining the number of colony-forming units (CFUs) after 18 h treatment with hymenoptaecin at five different abaecin concentrations plus the zero control. There was a wider range of responses over a 10-fold abaecin concentration range. Points show means ± s.d. (*n* = 10 for each point) and lines are optimized dose–response curves fitted to the raw data. Although hymenoptaecin was tested up to 10 μM, at higher concentrations growth was completely inhibited, so the *x*-axis is truncated at 5 μM to better visualize differences between hymenoptaecin dose–response curves at different concentrations of abaecin. However, all data including that at hymenoptaecin concentrations of 10 μM were used in model fitting and the production of the dose–response curves. Plots of dose responses for bactericidal activity including 95% highest posterior density intervals are presented in electronic supplementary material, figure S2*a*.

Abaecin was originally isolated from the haemolymph of the European bumblebee *B. pascuorum* following immunization with *E. coli*, and its antibacterial activity was demonstrated against this species [46]. However, we were unable to detect any antibacterial activity against *E. coli* strain D31 at concentrations ranging from 20 µM (figure 1*a*) to 200 µM (data not shown). In the original report [46], the authors used *E. coli* strain D22, which has a defective cell envelope. We therefore hypothesized that abaecin may need a compromised cell envelope or the presence of a pore-forming peptide to gain access to its intracellular target(s) and/or that its biological function may be to reduce the minimum inhibitory concentration (MIC) of other antibacterial peptides. We therefore tested abaecin in the presence of a sublethal dose (1.3 µM) of bumblebee hymenoptaecin, a glycine-rich AMP that is thought to form pores in the bacterial envelope [57]. As expected, 1.3 µM hymenoptaecin did not show any antibacterial activity alone (figure 1*a*). However, the simultaneous application of 1.3 µM hymenoptaecin and 20 µM abaecin completely suppressed the growth of *E. coli* D31 cells (figure 1*a*). These data suggest that sublethal concentrations of hymenoptaecin may either compromise the bacterial envelope, allowing abaecin to pass the membrane and gain access to its intracellular target(s), or reduce the MIC of hymenoptaecin against Gram-negative bacteria.

### (b) Abaecin potentiates the antibacterial activity of hymenoptaecin

To investigate whether abaecin can reduce the MIC of other peptides, we tested the quantitative antibacterial effects of abaecin and hymenoptaecin alone and in combination using *E. coli* 498 cells as a model to estimate growth parameters and their probability distributions (see electronic supplementary material). When bumblebee hymenoptaecin was applied in isolation, the bacteriostatic IC_50_ value (estimated concentration for 50% growth inhibition) was 1.88 μM, with 95% highest posterior density intervals of 1.58–2.18 µM. By contrast, *B. terrestris* abaecin had no inhibitory effect at any of the concentrations we tested (electronic supplementary material, figure S1). However, dose–response curves calculated for hymenoptaecin at each abaecin concentration when the two peptides were applied together showed that the combination had a significantly greater inhibitory effect than hymenoptaecin alone, with the bacteriostatic IC_50_ value for hymenoptaecin falling to approximately 0.8–1.1 μM at all concentrations of abaecin (electronic supplementary material, figures S1 and S2, and table S2).

We also determined the impact of the AMPs on bacterial cell survival. Bumblebee hymenoptaecin in isolation reduced cell viability to zero at concentrations of 5 and 10 μM (figure 1*b*) and the bactericidal IC_50_ value (estimated concentration for 50% loss of viability) was found to be 3.01 μM, with 95% highest posterior density intervals of 2.54–3.59 µM. As observed in the growth inhibition assays, abaecin alone was found to have no impact on cell viability at any of the concentrations tested up to 20 µM (figure 1*b*). However, once again we found that the combination of hymenoptaecin and abaecin had a significantly greater impact on viability than hymenoptaecin alone, with the bactericidal IC_50_ values for hymenoptaecin falling to between approximately 1.8 and approximately 0.6 μM in a dose-dependent manner as the concentration of abaecin increased (figure 1*b*; electronic supplementary material, figure S2 and table S2).

### (c) Hymenoptaecin and abaecin cause structural changes on the bacterial cell surface

**Figure 2.**
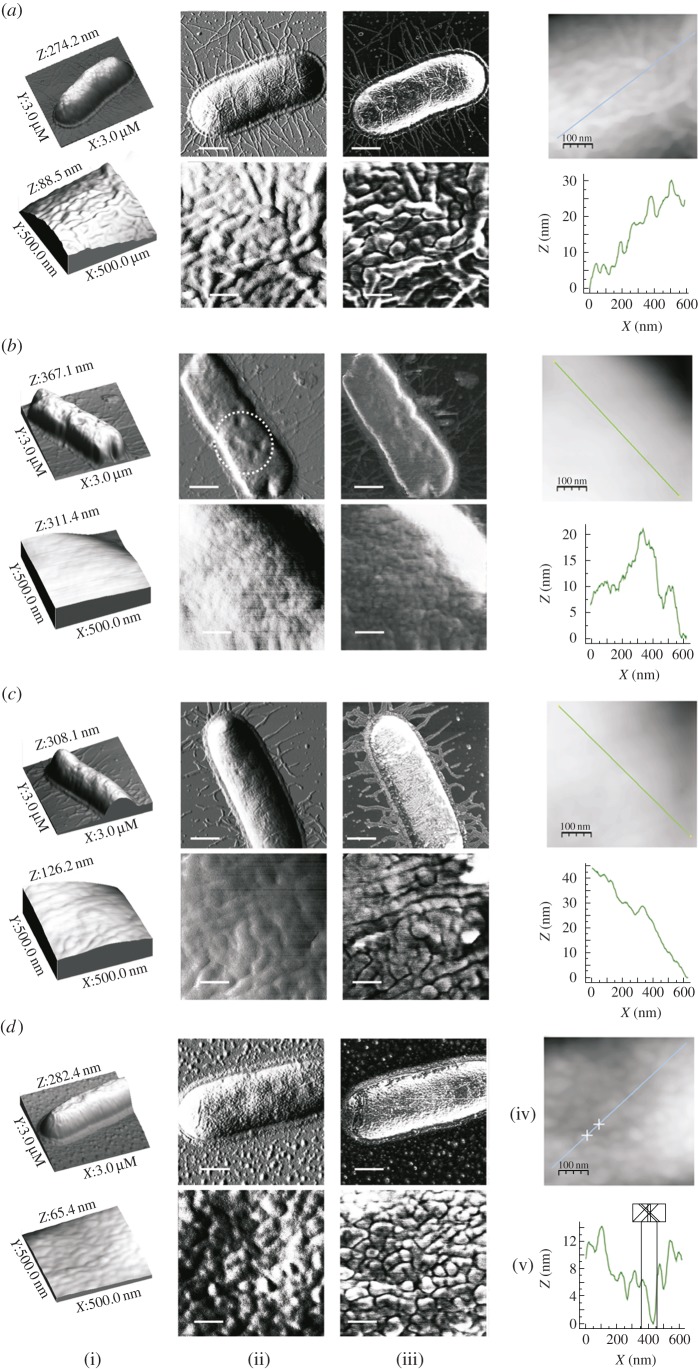
Gross morphology and surface properties of *E. coli* JM83 cells treated with abaecin and hymenoptaecin, alone and in combination. The figure shows the bacteria incubated without AMPs (control) (*a*) and in the presence of 0.5 μM hymenoptaecin (*b*) or 20 µM abaecin (*c*) or 0.5 μM hymenoptaecin plus 20 µM abaecin (*d*). In each panel, (i) is the three-dimensional image, (ii) is the peak force error, (iii) is the deformation image of the bacteria, (iv) shows height images of the bacterial cell surface and (v) shows section profiles corresponding to the lines marked in (*d*). Scale bars = 600 nm for the upper panels in (ii) and (iii), and 100 nm for (iv) and the lower panels in (ii) and (iii). (Online version in colour.)

The exposure of *E. coli* cells to hymenoptaecin and abaecin, alone or in combination, caused considerable changes to the bacterial cell surface as determined by atomic force microscopy (AFM) imaging. Untreated bacteria retained their normal rod-shaped appearance, with easily distinguishable envelopes and flagella, and the envelope surface was decorated with regularly spaced small granules and irregular long grooves (figure 2*a*) as previously described [53]. Cells exposed to hymenoptaecin were characterized by a highly unusual morphology, with a lumpy and irregular cell shape and fewer and ill-defined flagella (figure 2*b*). The cell surface was less granular than the control cells and featured numerous irregular cavities (encircled by a dotted line in figure 2*b*). The surfaces of bacteria treated with abaecin were smoother than the control cells (figure 2*c*). The granules and grooves were visible although less pronounced, and there were fewer flagella, but as stated above this had no impact on cell growth or viability. Unlike either of the effects described above, the combined treatment with abaecin and hymenoptaecin produced cells that appeared normal in shape but highly abnormal in structure, with a disrupted envelope and missing flagella (figure 2*d*). The surface was covered with smaller but more numerous and pronounced granules surrounded by recesses 3–6 nm in depth and up to 100 nm wide. The cells were also surrounded by many small structures, probably representing damaged and detached parts of the envelope (figure 2*d*). Despite these visually distinct and treatment-specific properties, we detected no significant differences in the cell-surface RMS roughness values between cells exposed to the combined peptides and untreated controls (electronic supplementary material, table S3).

### (d) Hymenoptaecin acts by perforating the bacterial envelope

**Figure 3.**
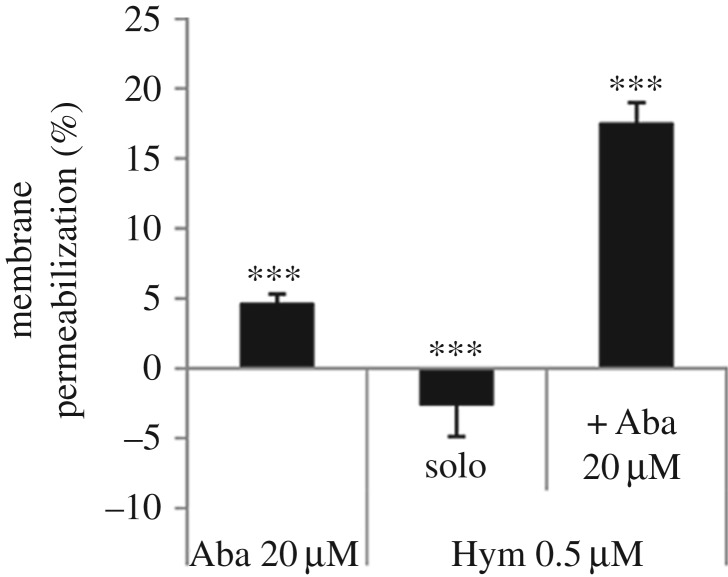
Membrane permeabilization assay in *E. coli* JM83 cells showing the activities of abaecin (Aba) and hymenoptaecin (Hym) against the bacterial cell envelope determined by measuring the β-galactosidase leaking into the medium. Cells in mid-logarithmic phase were treated with peptides alone and in combination and the absorbance was measured at 405 nm, proportional to the amount of released β-galactosidase. Living bacteria incubated with no AMPs were used as a negative control and bacteria killed by treatment with 5 µM synthetic cecropin B (Sigma) were used as a positive control. After subtracting the perforation level of the negative control from all measurements, the perforation level of the dead bacteria was set to 100%. Values represent means ± s.d. (*n* = 3). Statistical significance versus control: ***p < 0.001.

To gain insight into the mechanisms of action of abaecin and hymenoptaecin, we carried out a cell permeabilization assay using *E. coli* strain JM83, which constitutively expresses cytoplasmic β-galactosidase. As suggested by the growth inhibition assays (figure 1*a*), we found that abaecin perforated the bacterial envelope weakly, with the highest concentration (20 µM) achieving approximately 4.8% perforation compared with untreated control cells (figure 3; electronic supplementary material, figure S3*a*). Hymenoptaecin did not perforate the bacterial membrane at a concentration of 0.5 µM (figure 3) but showed dose-dependent perforation rates of 19% and 25% at concentrations of 0.9 and 1.4 µM, respectively (electronic supplementary material, figure S3*b*). However, the combination of 0.5 µM hymenoptaecin and 20 µM abaecin increased the perforation rate to 18% (figure 3).

### (e) Abaecin interacts with the *Escherichia coli* chaperone DnaK

**Figure 4.**
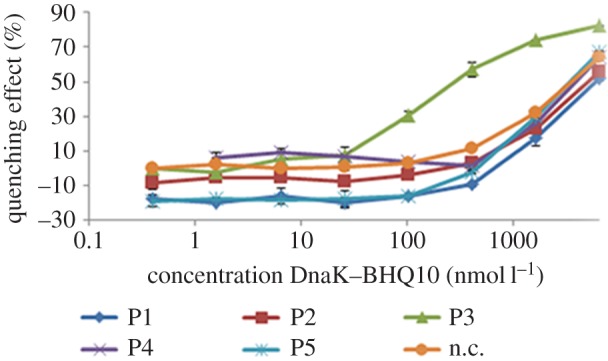
Quenching effect for different proline-rich AMPs. The quenching curves for metalnikowin-I (P1), metalnikowin-IIA (P2), abaecin (P3), metchnikowin-1 (P4), metchnikowin-2 (P5) and negative control (n. c.) are shown. Only the DnaK-binding curve of *B. pascuorum* abaecin was sigmoidal. Values represent means ± s.d. (*n* = 3).

To identify potential intracellular targets of abaecin, we used our recently developed peptide–protein interaction assay based on the measurement of fluorescence resonance energy transfer (FRET) between a fluorescein-labelled proline-rich peptide and a quencher-labelled bacterial DnaK probe, DnaK–BHQ10 [51]. We also included four other proline-rich AMPs, namely metalnikowin I and IIA from *Palomena prasina* and metchnikowin 1 and 2 from *Drosophila melanogaster* (electronic supplementary material, table S4), to provide further data concerning the mechanism of action. We measured the quenching effect of each AMP against the probe DnaK–BHQ10 compared to the effect of a control peptide, apidaecin 1b (#9–18) [Cf-PQPRPPHPRL-OH]**,** which interacts minimally with DnaK [51]. This minimal binding explains the small increase in control readouts with increasing probe concentration (figure 4).

Only the DnaK-binding curve of *B. pascuorum* abaecin was sigmoidal, confirming its interaction with DnaK with a maximum quenching effect of 74% (figure 4). The disassociation values (*K*_d_) were determined by nonlinear regression, with the optimal value of 0.19 µmol ^−1^ for abaecin (electronic supplementary material, table S4). Abaecin thus compares well to the reported *K*_d_ values of other DnaK-binding proline-rich peptides such as native oncocin and pyrrhocoricin derivatives (approx. 0.1 µmol l^−1^ [51]). It was not possible to assign a *K*_d_ value to metalnikowin I or IIA or metchnikowin 1 or 2 (electronic supplementary material, table S4), suggesting these peptides do not interact significantly with bacterial DnaK.

### (f) Abaecin possesses an atypical DnaK-binding element

**Figure 5.**
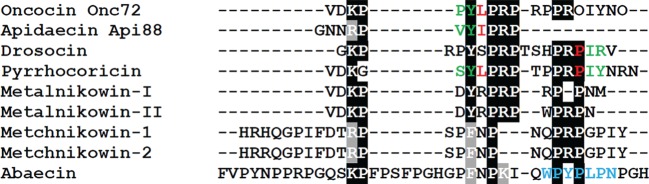
Sequence comparison of different proline-rich AMPs. Amino acid residues that occupy the central pocket of DnaK [58] are shown in red. Residues that occupy the –1 and –2 binding sites are shown in green. The putative DnaK-binding sequence of *B. pascuorum* abaecin is shown in blue. The alignment was generated with ClustalW and manually edited for the improved alignment of proline (P) residues. Ornithine residues in the artificial peptide oncocin Onc72 are shown as O.

We compared the amino acid sequences of abaecin, the metalnikowins and metchnikowins with four other proline-rich DnaK-binding AMPs (oncocin Onc72, apidaecin Api88, drosocin and pyrrhocoricin) in order to determine the functional sequence that interacts with DnaK (figure 5). These four AMPs have recently been co-crystallized with the substrate-binding domain of DnaK in order to determine their binding mechanism [58]. The alignment of abaecin with these four peptides showed that despite its 74% binding efficacy (figure 4), abaecin possesses neither a conserved YL/IPRP motif nor a sequence that favours binding in the reverse mode (figure 5). We therefore analysed abaecin using the limbo server (http://limbo.switchlab.org; [59]) and found that the sequence WPYPLPN was the best-scoring DnaK-binding sequence (score 4.27). This is unique among the known DnaK-binding sequences in terms of its amino acid composition.

## 4. Discussion

We report that insect AMPs with two distinct mechanisms of action can functionally interact resulting in greater combined antibacterial activity than either can achieve in isolation. Specifically, abaecin and hymenoptaecin from the bumblebee species *B. pascuorum* and *B. terrestris* were tested alone and in combination. Although hymenoptaecin (identical in both species) showed dose-dependent bacteriostatic and bactericidal activity at concentrations above 2 µM, abaecin alone (differing by one residue between the species) had no impact on bacterial growth or survival even at a concentration of 200 µM, which is far higher than the typical physiological concentration of AMPs in the insect haemolymph. Nevertheless, when the AMPs were applied simultaneously, they achieved absolute inhibition of growth and 100% lethality at concentrations of 1.3 µM hymenoptaecin and 20 µM abaecin. More detailed quantitative analysis confirmed that hymenoptaecin applied in isolation had a bacteriostatic IC_50_ value of 1.88 μM (95% highest posterior density 1.58–2.18 μM) and a bactericidal IC_50_ value of 3.01 μM (95% highest posterior density 2.54–3.59 μM). In the presence of 1.25–20 μM abaecin, the bacteriostatic IC_50_ value of hymenoptaecin fell to approximately 0.8–1.1 μM, the narrow range suggesting a near ‘on–off’ impact on bacterial growth when both AMPs were presented simultaneously. By contrast, the bactericidal IC_50_ value of hymenoptaecin fell to approximately 1.75 μM in the presence of 1.25 μM abaecin and to approximately 0.63 μM in the presence of 20 μM abaecin, the broader range indicating a cooperative dose-dependent effect on bacterial cell viability influenced by the concentration of both AMPs. A comparable observation has been reported for the silkworm moth (*Bombyx mori*) peptides lebocin 3 and cecropin D, in which the combination of both peptides reduced the MIC values compared with the individual peptides [16].

Our growth inhibition and cell viability assay results were supported by AFM imaging of the bacterial cell and envelope surface. In isolation, each AMP had a visible but distinct influence on the external morphology of the cells, in each case changing the surface properties of the cell envelope and the appearance of the flagella in a specific manner. Abaecin appeared to have no significant impact on gross morphology, whereas hymenoptaecin caused the cell to lose its regular rod-like shape and become lumpy and rugged, suggesting a more severe impact concordant with its dose-dependent bacteriostatic and bactericidal effects. The molecular basis of interactions between AMPs and the bacterial envelope can be complex because they depend on the properties of both the peptide and the cell envelope itself, thus determining the antimicrobial spectrum of each AMP [60]. When both peptides were present, the morphology of the cell was extensively disrupted and the presence of external structures indicated that the integrity of the cell had been breached, agreeing with the growth inhibition and cell viability assays, in which low concentrations of both AMPs applied simultaneously caused growth arrest and cell death.

We next looked at the mechanism of action for each AMP. Hymenoptaecin is a glycine-rich polypeptide that disrupts the bacterial membrane and occupies a place along the antibacterial spectrum somewhere between the groups of helical, ionophoric peptides (such as cecropin A) with activity against all bacteria, and those preferentially acting against either Gram-positive or Gram-negative species [57]. Previous reports have suggested that hymenoptaecin sequentially makes the *E. coli* outer and inner membranes permeable [57]. In Gram-negative bacteria such as *E. coli*, most cationic defence peptides interact first with negatively charged lipopolysaccharides on the outer membrane to reach the inner membrane, displacing the divalent ions that control the stability of the envelope [60,61]. We therefore carried out an assay for cell permeability, in which intracellular β-galactosidase leaks from an indicator strain of *E. coli* if membrane integrity is lost. The permeability of the membrane was only marginally increased by abaecin alone, but we observed a dose-dependent increase in permeability in response to higher concentrations of hymenoptaecin, confirming that the bacteriostatic and bactericidal activity of this peptide reflects its ability to perforate the bacterial envelope.

Short, proline-rich AMPs are usually translocated across membranes in a non-lytic manner because the positive charge enhances their interaction with the membrane surface [62] allowing them to move first into the periplasmic space and then into the cytoplasm by irreversibly interacting with a docking receptor or transporter, or the 60-kDa bacterial chaperone GroEL. We propose that bumblebee abaecin functions by reducing the MIC of other AMPs and/or by inhibiting the molecular chaperone DnaK, but our data and other reports [46] suggest it cannot cross an intact cell envelope. Apidaecins are also known to lack this ability and therefore require a compromised envelope to enter the cell [44]. Once the AMP reaches the cytoplasm, it may bind to a ribosome, inhibiting protein synthesis, or to DnaK, inhibiting protein folding [42,43,63,64]. The proline residues restrict the flexibility of the peptide and may therefore reduce the loss of entropy when it binds to DnaK. DnaK recognizes extended peptide constituents as well as positively charged residues within and outside its substrate-binding cleft [65].

We used our recently developed FRET-based peptide–protein interaction assay to confirm that abaecin interacts with DnaK, but alignment with other proline-rich peptides revealed that it lacks any known DnaK-binding motif. Normally, the characteristic sequence stretch YL/IPRP is positioned in the DnaK substrate-binding cleft, with a leucine or isoleucine residue occupying the central hydrophobic pocket [58]. Additional hydrophobic binding sites preceding the central pocket are occupied by the conserved tyrosine residue and another, preferentially hydrophobic amino acid residue, defined as positions –1 and –2, respectively. Some proline-rich AMPs can bind DnaK in the reverse orientation, independent of the YL/IPRP motif. For example, a proline residue near the C-terminus of drosocin occupies the central pocket, whereas the subsequent residues (isoleucine and arginine) are orientated in the –1 and –2 binding sites. The use of artificial peptides has unambiguously shown that the replacement of the aliphatic leucine or isoleucine residue within the drosocin YL/IPRP motif with a hydrophilic serine residue prevents binding to DnaK in the forward mode. Pyrrhocoricin can bind to DnaK in both of the above orientations, the forward mode mediated by the canonical YLPRP sequence, and the reverse mode mediated by the PIY sequence located in the central pocket, –1 and –2 binding sites, respectively.

The features discussed above explain the failure of metalnikowins and metchnikowins to bind DnaK. In the metalnikowin sequences, a positively charged arginine residue replaces the leucine/isoleucine residue in the YL/IPRP motif and a negatively charged aspartic acid residue preceding the conserved tyrosine is likely to restrict binding to the DnaK substrate cleft even further. In the metchnikowin sequences, the YL/IPRP motif is even less conserved, with a hydrophilic asparagine residue at the leucine/isoleucine position. Although predictions are difficult because of the limited number of peptides analysed thus far, it appears unlikely from the available data that metalnikowins and metchnikowins bind with high affinity to DnaK in the reverse mode. The experimental data from our FRET-based quenching assay confirm that metalnikowins and metchnikowins do not bind to DnaK in either mode (figure 4).

As stated above, abaecin binds DnaK with up to 74% efficacy without the benefit of a conserved YL/IPRP motif or a reverse binding mode sequence (figure 4). The limbo prediction software package [59] instead predicts that WPYPLPN is the best-scoring sequence for DnaK binding (score 4.27). This atypical sequence, comprising alternating proline and aromatic residues (tryptophan or tyrosine) and the bulky aliphatic residue leucine, should adopt a comparatively rigid and hydrophobic structure and should therefore be the key determinant that allows abaecin to bind DnaK.

Based on the results of our molecular and functional assays, it therefore seems apparent that the functional interaction between hymenoptaecin and abaecin is derived from the ability of hymenoptaecin to create pores that allow abaecin to enter the cell and interact with DnaK, as has been shown for other small hydrophobic molecules [57]. In the absence of abaecin, higher concentrations of hymenoptaecin inhibit and eventually kill bacterial cells probably because more extensive perforation of the envelope causes the leakage of electrolytes, but much lower levels of perforation are sufficient to kill the cells, if abaecin is also present. In this model, abaecin potentiates the activity of hymenoptaecin and in return hymenoptaecin enables the diffusion of abaecin through the bacterial cell membrane. In the permeabilization assay, the rate of perforation increased in the presence of abaecin, i.e. 0.5 μM hymenoptaecin was insufficient to increase the permeabilization rate above the control level, but in the presence of abaecin the permeabilization rate increased to 18% (which required 0.9 μM of hymenoptaecin acting alone). Similarly, the sensitivity of *E. coli* towards *Hyalophora cecropia* cecropin B was enhanced following treatment with *H. cecropia* attacin, which affects the permeability of the *E. coli* outer membrane [15], and the negligible antibacterial facticity of *B. mori* lebocin 3 presented alone was increased by the addition of the cell-permeabilizing detergent Triton X-100 [16].

Abaecin may also affect the bacterial membrane directly to a small extent, based on the observation that both versions of the peptide can increase the permeability of bacterial cells marginally, perhaps owing to their basic but hydrophilic nature, as clearly shown in hydropathy plot (electronic supplementary material, figure S4). The hydrophilic nature of abaecin may promote the initial interaction between hymenoptaecin and the bacterial membrane, followed by a stronger direct interaction involving the basic and hydrophobic residues of hymenoptaecin, which may promote intercalation. However, a more likely explanation is that abaecin indirectly contributes to the increased permeability of the bacterial envelope by interfering with basic metabolism and housekeeping functions, thereby preventing the integrity of the membrane from being maintained. We propose that hymenoptaecin facilitates the activity of abaecin by allowing it to gain access to the cell, where it interacts with DnaK and inhibits normal housekeeping functions, resulting in an exacerbated loss of membrane integrity in addition to the pores formed by hymenoptaecin. This would also explain the distinct morphology and surface attributes of cells exposed to hymenoptaecin and those simultaneously exposed to both peptides. However, this is a model based on our observations and neither the facilitated accumulation of abaecin in the presence of hymenoptaecin nor the antimicrobial activity of intracellular abaecin have been confirmed directly.

## 5. Conclusion

We have shown that abaecin potentiates the activity of hymenoptaecin, and hymenoptaecin in turn facilitates the activity of abaecin against Gram-negative bacteria. Specifically, abaecin interacts with the bacterial chaperone DnaK, but requires the pore-forming action of AMPs such as hymenoptaecin before it can penetrate the membrane and gain access to its intracellular target(s), which is necessary to exert its full activity. However, we cannot exclude the possibility that the potentiation might only be owing to the increase of membrane disruptive activity. Insects may be able to defend themselves against a wider range of microbial challenges by exploiting the more efficient innate immunity achieved through such functional interactions, which may reduce the cost of defence by minimizing trade-offs with other components of the immune system and increase the diversity and/or specificity of responses from a limited AMP repertoire. Furthermore, because the antimicrobial properties of abaecin and hymenoptaecin are based on relatively short peptide sequences, it should be possible to synthesize combined artificial peptides and test their potential against pathogenic bacteria, where such interactive effects may boost their therapeutic efficacy.

## Supplementary Material

Supporting Information

## Supplementary Material

Supplementary Tables and Figures

## Data accessibility

Electronic supplementary material is available online.
