# Supplementary material for: Insect antimicrobial peptides show potentiating functional interactions against Gram-negative bacteria
Source: Proc Biol Sci. 2015 May 7;282(1806):20150293. doi: 10.1098/rspb.2015.0293 (PMC4426631; doi:10.1098/rspb.2015.0293)
Supplement: Supporting Information [file rspb20150293supp1.pdf]

Insect antimicrobial peptides show potentiating functional interactions against Gram-negative bacteria

Mohammad Rahnamaeian[1,†], Małgorzata Cytryńska[2,†], Agnieszka Zdybicka-Barabas[2], Kristin Dobslaff[3], Jochen Wiesner[1], Richard M Twyman[1,4], Thole Züchner[3], Ben M Sadd[5], Roland Regoes[6], Paul Schmid-Hempel[6], Andreas Vilcinskas[1,7*]

[1] Department of Bioresources, Fraunhofer Institute for Molecular Biology and Applied Ecology, Winchester Strasse 2, D-35394 Giessen, Germany

[2] Department of Immunobiology, Institute of Biology and Biochemistry, Maria Curie-Sklodowska University, Akademicka St. 19, 20-033 Lublin, Poland

[3] Institute of Bioanalytical Chemistry, Faculty of Chemistry and Mineralogy and Center of Biotechnology and Biomedicine, University of Leipzig, Deutscher Platz 5, D-04103 Leipzig, Germany

[4] TRM Ltd, PO Box 93, York YO43 3WE, United Kingdom

[5] School of Biological Sciences, Illinois State University, Campus Box 4120 Normal, Illinois 61790, USA

[6] ETH Zürich, Institute of Integrative Biology, ETH-Zentrum CHN, Universitätsstrasse 16, CH-8092 Zürich, Switzerland

[7] Institute of Phytopathology and Applied Zoology, Justus-Liebig-University of Giessen, Heinrich-Buff-Ring 26-32, D-35392, Giessen, Germany

[†] Authors with equal contributions

[*] Corresponding author: Andreas.Vilcinskas@agrar.uni-giessen.de

Supporting Information

Materials and Methods:

i) Peptide synthesis and modification.

The peptide sequences shown in Tables *S1* and *S4* were selected according to their reported structural and functional properties, synthesized and purified to >95%. *B. terrestris* hymenoptaecin and abaecin were synthesized by Genscript (Piscataway, USA) and EZBiolabs (Carmel, USA), respectively. The mature peptide sequences were based on data from closely-related species [1] and EST information derived from *B. terrestris* [2]. Other peptides were synthesized by PANATecs (Tübingen, Germany). All peptides were lyophilized for storage, and were resuspended and diluted in double-distilled water, which was used as the zero peptide control. The metalnikowin I and IIA (*Palomena prasina*), abaecin (*Bombus pascuorum*), and metchnikowin 1 and 2 (*Drosophila melanogaster*) peptides were modified by the N-terminal addition of 5(6)-carboxyfluorescein and C-terminal amidation for the quenching assay. *Bombus pascuorum* hymenoptaecin was modified by the addition of an N-terminal pyroglutamate. The Black Hole Quencher 10 succinimidyl ester (BHQ10-NHS-ester, >75% purity) was obtained from BioCat (Heidelberg, Germany).

ii) *E. coli* permeabilization assay.

The membrane permeabilizing activities of AMPs were determined using *E. coli* strain JM83 on the basis of β-galactosidase activity leaking from the cytoplasm [3]. Peptides were pre-incubated for 15 min at 37°C in 23 µl 20 mM phosphate buffer (pH 6.8) and 2 µl of *E. coli* suspension containing 5×10^5 CFU of mid-logarithmic phase cells in the same buffer were added and incubated at 37°C for 45 min. The suspension was then mixed with 220 µl 20 mM HEPES/150 mM NaCl (pH 7.5) and 5 µl 50 mM aqueous *p*-nitrophenyl-β-D-galactopyranoside. The samples were incubated at 37°C for 90 min and the absorbance was measured at 405 nm, which is proportional to the amount of released β-galactosidase. Live bacteria incubated with medium only were used as a negative control and bacteria killed by treatment with 5 µM synthetic cecropin B (Sigma-Aldrich) were used as a positive control (100% permeabilization). Before setting the perforation level of the positive control as 100%, the perforation value obtained for the

negative control was subtracted from all other measurements. All assays were carried out three times, each time in triplicate. The results were presented as ±S.D. (n=3). The statistical analysis was performed using Student' *t* test. Statistical significance: *p<0.05, **p<0.01, ***p<0.001.

iii) AFM imaging of bacterial cells.

Hundred µl of log-phase *E. coli* JM83 cells (OD_{600} = 0.2) in LB medium were incubated for 1.5 h at 37°C with or without 20 µM abaecin and/or 0.5 µM hymenoptaecin. The samples were then centrifuged at 8000 × g for 10 min at 4°C, and the pellet was washed twice in 100 µl apyrogenic water. The bacteria resuspended in 5 µl apyrogenic water were applied to mica disks and dried overnight at 28°C before imaging. The cell surface was imaged in Analytical Laboratory, Faculty of Chemistry, UMCS, Lublin, Poland using a NanoScope V AFM (Veeco, USA. All measurements were carried out in "PeakForce QNM" mode using an NSG 30 silicon tip with a spring constant of 20 N/m (NT-MDT, Russia). The data were analyzed with Nanoscope Analysis software v1.40 (Veeco, USA). Three fields on each mica disk were imaged. Three dimensional images and section profiles were prepared using WSxM v5.0 software [4]. The roughness values were measured over the entire bacterial cell surface on 3×3 μm^2 areas. The average surface root-mean-square (RMS) roughness was calculated from 25 fields (300×300 nm^2). The data were analyzed using Statistica ver. 6 (StatSoft, Inc., Tulsa, OK, USA). Statistical significance was determined by ANOVA (Tukey's Honestly Significant Difference test; p<0.05).

iv) Determination of K_d values.

The dissociation constants (K_d values) were determined using the software package SlideWrite v7.01 (http://www.slidewrite.com). The quenching effects were plotted against the DnaK concentration (logarithmic abscissa). A non-linear regression using the dose-response logistical transition function of the program yielded K_d values, according to the formula

$$y = a0 + a1/(1 + x/a2)a3 \qquad \text{(eq. E1)}$$

where a0, a1, a2, and a3 are the fitting parameters with a2 being the K_D-value.

v) Coarse growth inhibition assays.

Bacteria in mid-logarithmic phase were used for all growth inhibition assays. The initial OD_{600} for all assays was set to 0.001 to ensure the full contact of each bacterial cell with added AMPs. The assays were carried out in 96-well plates (Griener Bio One, Frickenhausen, Germany) in an Eon™ Microplate Spectrophotometer (BioTek Instruments, VT, USA) for 16 h while recording of changes in the OD_{600} at 20-min intervals. For each assay, a control culture was included with medium only. The AMPs used in this assay are shown in Tables *S1* and *S4*.

vi) Dose response curve quantification

Growth inhibition assay. *E. coli* cells (DSM 498) were grown in Muller-Hinton broth (Sigma-Aldrich) for 14 h at 30ºC and counted using a FastRead 102 counting chamber (Immune Systems, UK). The suspension was adjusted in fresh medium to achieve a cell density of 5×10^4 per 80 µl. AMP treatments and controls were placed in the wells of a 96-well flat-bottomed plate. A treatment dilution matrix was created with 10 µl volumes of abaecin (final concentrations of 0, 1.25, 2.5, 5, 10, and 20 µM) in rows, and 10 µl volumes of hymenoptaecin (final concentrations of 0, 0.625, 1.25, 2.5, 5, and 10 µM) in columns. Individual treatment combinations were replicated five times on different plates, with the treatment matrix being rotated for each plate to reduce measurement bias. We added 80 µl of the bacterial suspension to each well. Plates were incubated at 30ºC in a shaking incubator. The OD_{600} was measured every hour for 12 consecutive hours using a SpectraMax M2^e microplate reader (Molecular Devices, Sunnyvale, USA). Bacteria-free calibration blanks were used to correct the OD_{600} values.

Cell viability. The same protocol described above was used for bacterial pre-culture, plate layout and cultivation. Each individual treatment combination was replicated ten times. Bacteria were grown for 18 h with AMPs before the number of viable cells was assessed. Well contents were serially diluted 1:50 to give a total of five dilutions. We then spread 10 µl of each dilution on Muller-Hinton medium (Sigma-Aldrich) with 3% agar, and incubated the plates overnight at 37ºC. Colony-forming units (CFUs), each representing a viable cell from the culture, were counted at a dilution where each individual CFU could be distinguished.

Data analysis for dose-response curves. All statistical analysis was carried out using R v2.11.1 for Mac. For each replicate of the OD temporal measurements, a spline-fitted growth curve was created and the maximum growth rate was calculated using *grofit* [7]. We used the following model to describe the relationships between AMP concentration *A*, and the growth rate, *g*:

$$g = r - (E_{max} A^h) / (A_{50}^h + A^h)$$

where *r* denotes the bacterial growth rate in the absence of AMPs, E_{max} the maximum effect at high concentrations, and A_{50} the AMP concentration at which 50% of the maximum effect is reached. The parameter *h* is the Hill coefficient determining how steeply the effect increases around the concentration A_{50}. This model was fitted to the maximum growth rates with a Markov chain Monte Carlo algorithm using Just Another Gibbs Sampler (8) in combination with the R-package rjags (9). A model of the same format was fitted to the cell viability data. Parameter estimates for AMP concentrations resulting in 50% inhibition of growth and 50% reduction in viable cells were derived, along with 95% highest posterior density intervals for these estimates.

References:

1. Rees JA, Moniatte M, Bulet P. 1997 Novel antibacterial peptides isolated from a European bumblebee, Bombus pascuorum (Hymenoptera, Apoidea). Insect Biochem. Mol. Biol. **27(5)**, 413-422.

2. Sadd BM, Kube M, Klages S, Reinhardt R, Schmid-Hempel P. 2010 Analysis of a normalised expressed sequence tag (EST) library from a key pollinator, the bumblebee Bombus terrestris. BMC Genomics **11**, 110. (doi: 10.1186/1471-2164-11-110)

3. Zdybicka-Barabas A, Mak P, Klys A, Skrzypiec K, Mendyk E, Fiołka MJ, Cytryńska M. 2012 Synergistic action of Galleria mellonella anionic peptide 2 and lysozyme against Gram-negative bacteria. Biochim. Biophy. Acta **1818(11)**, 2623-2635.

4. Horcas I, Fernández R, Gómez-Rodríguez JM, Colchero J, Gómez-Herrero J, Baro AM. 2007 WSXM: a software for scanning probe microscopy and a tool for nanotechnology. Rev. Sci. Instrum. **78(1)**, 013705.

5. Chernysh S, Cociancich S, Briand J-P, Hetru C, Bulet P. 1996 The inducible antibacterial peptides of the Hemipteran insect *Palomena prasina*: Identification of a unique family of proline rich peptides and of a novel insect defensin. J. Insect Physiol. **42(1)**, 81-89.

6. Levashina EA, Ohresser S, Bulet P, Reichhart JM, Hetru C, Hoffmann JA. 1995 Metchnikowin, a novel immuneinducible prolin-rich peptide from *Drosophila* with antimicrobial and antifungal properties. Eur. J. Biochem. **233**, 694-700.

7. Kahm M, Hasenbrink G, Lichtenberg-Frate H, Ludwig J, Kschischo M. 2010 grofit: Fitting Biological Growth Curves with R. J. Stat. Softw. **33(7)**, 1-21.

8. Plummer M. 2003 JAGS: A Program for Analysis of Bayesian Graphical Models Using Gibbs Sampling, Proceedings of the 3rd International Workshop on Distributed Statistical Computing (DSC 2003), March 20–22, Vienna, Austria. ISSN 1609-395X.

9. Plummer M. 2014 rjags: Bayesian graphical models using MCMC. R package version 3-13. http://CRAN.R-project.org/package=rjags
