# Supplementary material for: Insect antimicrobial peptides show potentiating functional interactions against Gram-negative bacteria
Source: Proc Biol Sci. 2015 May 7;282(1806):20150293. doi: 10.1098/rspb.2015.0293 (PMC4426631; doi:10.1098/rspb.2015.0293)
Supplement: Supplementary Tables and Figures [file rspb20150293supp2.pdf]

Supplementary Tables and Figures:

Table S1. Amino acid sequences of abaecin and hymenoptaecin tested in this study. Hymenoptaecin was N-terminally modified with pyroglutamate.

Peptide	Origin	Sequence	Reference
Hymenoptaecin	*Bombus pascuorum*	{Pyr}HADPQGSLVINGKKPLS	[1,2]
		GPDRRPSLDVDYHQRVYDRN	
	B. terrestris	GMNADAYGGLNIRPGQPAQP	
		HLGVQIQREYKNGFIRGYSQA	
		ERGPGGRISPSFGVGGGFRF	
Abaecin	*B. terrestris*	FVPYNPPRPGQSKPFPTFPGHG	[2]
		PFNPKIQWPYPLPNPGH	
Abaecin	*B. pascuorum*	FVPYNPPRPGQSKPFPSFPGHG	[1]
		PFNPKIQWPYPLPNPGH	

Table S2. Hymenoptaecin concentration estimates at different abaecin concentrations causing (A) 50% inhibition of bacterial growth and (B) 50% reduction in cell viability after 18 h. 95% Highest Posterior Density (HPD) intervals are given for each parameter estimation.

	(A) Hymenoptaecin concentration for 50% growth inhibition			(B) Hymenoptaecin concentration for 50% reduction in cell viability		
Abaecin (μM)	Estimate (μM)	Upper 95%HPD	Lower 95%HPD	Estimate (μM)	Upper 95%HPD	Lower 95%HPD
0	1.88	2.18	1.58	3.01	3.59	2.54
1.25	1.13	1.24	0.98	1.75	2.10	1.42
2.5	1.09	1.26	0.88	0.95	1.08	0.82
5	0.97	1.12	0.80	0.92	1.06	0.78
10	0.86	1.05	0.69	0.91	1.07	0.75
20	0.83	1.01	0.68	0.63	0.66	0.60

Table S3. The effect of AMPs on the roughness of *E. coli* JM83 cells.

	**Control**	**Hymenoptaecin (0.5 μM)**	**Abaecin (20 μM)**	**Hymenoptaecin + Abaecin**
**Roughness RMS value (nm; ±SD)**	7.265 (±1.876)	9.784 (±4.868)*	5.789 (±3.032)*	6.272 (±2.462)

Table S4. Sequences of different proline-rich peptides modified with N-terminal 5(6)-carboxyfluorescein (Cf) or by C-terminal amidation (NH_2). The calculated K_d values are based on the quenching assay. No K_d values could be determined for metalnikowin I, metalnikowin IIA, metchnikowin 1 or metchnikowin 2.

**Peptide**	**Origin**	**Sequence**	**Reference**	**K_d (µmol/L)**
Metalnikowin I (P1)	*Palomena prasina*	Cf-VDKPDYRPRPRPPNM-NH_2	[5]	> 5
Metalnikowin IIA (P2)	*Palomena prasina*	Cf-VDKPDYRPRPWPRPN-NH_2	[5]	> 5
Abaecin (P3)	*Bombus pascuorum*	Cf-FVPYNPPRPGQSKPFPSFPGHGPFNPKIQWPYPLPNPGH-NH_2	[1]	0.19 ± 0.03
Metchnikowin-1 (P4)	*Drosophila melanogaster*	Cf-HRHQGPIFDTRPSPFNPNQPRPGPIY-NH_2	[6]	> 5
Metchnikowin-2 (P5)	*Drosophila melanogaster*	Cf-HRRQGPIFDTRPSPFNPNQPRPGPIY-NH_2	[6]	> 5

Fig. S1

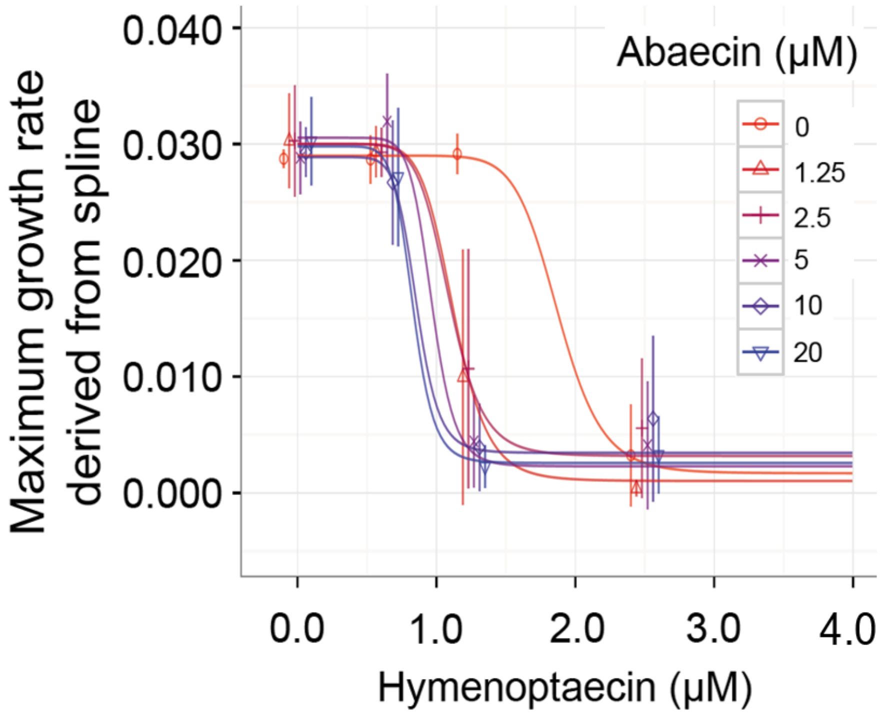

Fig. S1. *E. coli* growth rate. *E. coli* growth rates calculated from spline-fitted growth curves with optimized dose-response curves for abaecin alone and for hymenoptaecin at five different abaecin concentrations plus the zero control. There is a narrow range of responses over a 10-fold abaecin concentration range. Points show means ± SD (n = 5). Although hymenoptaecin was tested up to 10 µM, at higher concentrations growth was completely inhibited, so the x-axis is truncated at 4 µM to better visualise differences between hymenoptaecin dose-response curves at different concentrations of abaecin. However, all data including that at hymenoptaecin concentrations of 5 µM and 10 µM were used in model fitting and the production of the dose-response curves. Plots of dose responses for bacteriostatic activity including 95% highest posterior density intervals are presented in Figure S2B.

Fig. S2

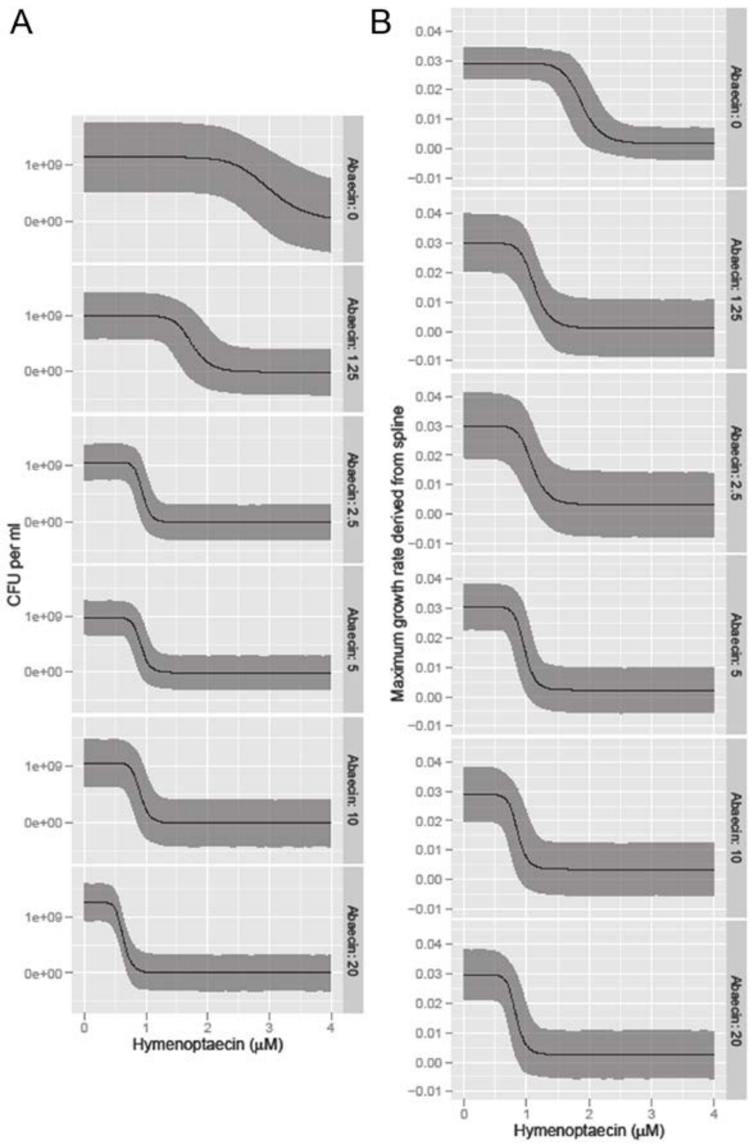

Fig. S2. Dose response curves with 95% highest posterior density intervals for parameter estimates of bactericidal activity (A) and bacteriostatic activity (B). Plots show estimated dose response curves across hymenoptaecin concentrations for, from top to bottom, abaecin concentrations of 0, 1.25, 2.5, 5, 10 and 20 μM, respectively. Although hymenoptaecin was tested up to 10 μM, at higher concentrations growth was completely inhibited, so the x-axis is truncated at 4 μM to better visualise differences between hymenoptaecin dose-response curves at different concentrations of abaecin. However, all data including that at hymenoptaecin concentrations of 5 μM and 10 μM were used in model fitting and the production of the dose-response curves.

Fig. S3

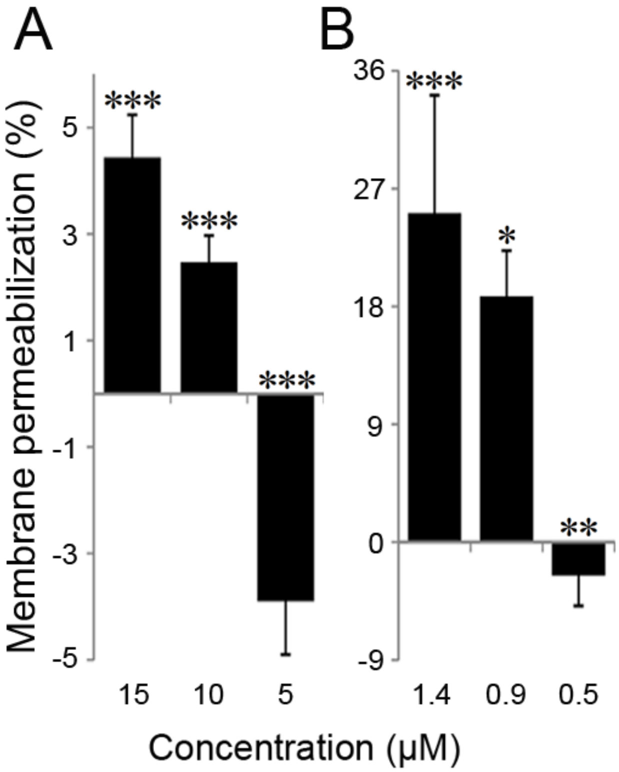

Fig. S3. Membrane permeabilization assay in *E. coli* JM83 cells showing the activities of abaecin (A) and hymenoptaecin (B) determined by measuring β-galactosidase leaking into the medium. Cells in mid-logarithmic phase were treated with AMPs and the absorbance at 405 nm was measured, which is proportional to the amount of released β-galactosidase. Living bacteria incubated with no AMPs were used as a negative control and bacteria killed by treatment with 5 µM synthetic cecropin B were used as a positive control. After subtracting the perforation level of the negative control from all measurements, the perforation level of the dead bacteria was set to 100%. Values represent means ± SD (n = 3). Statistical significance versus control: *$p<0.05$; **$p<0.01$; ***$p<0.001$.

Fig. S4

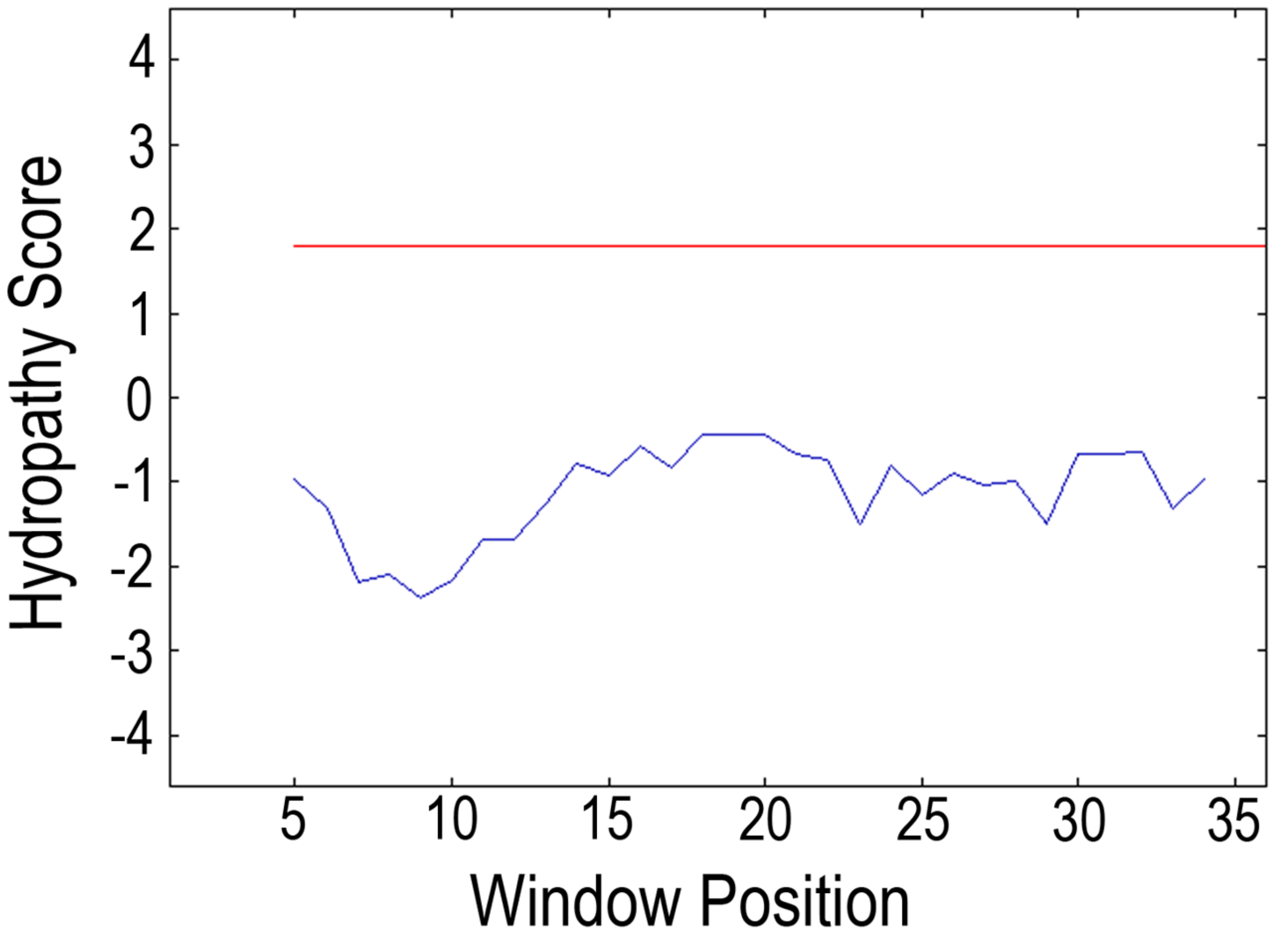

Fig. S4. Kyte Doolittle hydropathy plot for abaecin. Query statistics: window size = 9; start position = 1; end position = 39; query length = 39; effective length = 31. The Window Position values shown on the x-axis of the graph reflect the average hydropathy of the entire window, with the corresponding amino acid as the middle element of that window. The horizontal axis is scaled to include only those amino acids for which a windowed hydropathy score is computed. Peaks with score greater than 1.8 (red line) indicate possible transmembrane regions. The hydropathy score of each amino acid is shown in blue.
